# Self-Esteem and Body Satisfaction After Radical Orchiectomy With or Without Testicular Prosthesis: A Retrospective Study at a Mexican Tertiary Center

**DOI:** 10.7759/cureus.107889

**Published:** 2026-04-28

**Authors:** Juan C Cardona Contreras, Juan E Sanchez Nunez, Emmanuel Rosas-Nava, Jorge Jaspersen Gastelum, Hugo A Manzanilla García, Anuar D Berber Deseusa, Edson D Rodriguez Valle

**Affiliations:** 1 Urology, Hospital General de México “Dr. Eduardo Liceaga”, Mexico City, MEX; 2 Urology, The American British Cowdray Medical Center, Institución de Asistencia Privada, Mexico City, MEX; 3 Urology, Hospital General de México, Mexico City, MEX

**Keywords:** patient satisfaction, prosthesis, radical orchiectomy, self-esteem, testicular

## Abstract

Background: Radical orchiectomy remains the standard treatment for localized testicular cancer. However, the resulting anatomical loss may negatively affect body image and self-esteem, especially in young men. The use of testicular prostheses has been proposed as a reconstructive option to mitigate these psychosocial effects, though evidence on its impact on global self-esteem remains limited, particularly in Latin American populations.

Methods: We conducted a retrospective, analytical study among adult patients with non-metastatic testicular cancer who underwent radical orchiectomy at a tertiary public hospital in Mexico between 2019 and 2024. Participants were categorized into two groups based on whether they received a testicular prosthesis. This retrospective study assessed psychosocial outcomes following radical orchiectomy, using telephone-administered Rosenberg Self-Esteem Scale assessments and a study-specific, non-validated satisfaction questionnaire. Comparative analysis was performed using the Student’s t-test, with p<0.05 considered statistically significant.

Results: Of 110 eligible patients, 92 completed follow-up surveys (41 with prosthesis, 51 without). Patients with prostheses had significantly higher self-esteem scores (mean 31.8 vs. 23.6; p = 0.003). Global satisfaction with the implant was 100%, and 96% of recipients would undergo the procedure again. Dissatisfaction was most commonly reported for implant position (29%) and consistency (51%).

Conclusions: Immediate testicular prosthesis placement after orchiectomy may improve self-esteem and body satisfaction in testicular cancer survivors. These findings support the inclusion of prosthetic counseling as a routine component of surgical care, especially in health systems where reconstructive options are underutilized.

## Introduction

Testicular cancer is the most common solid malignancy among young adult males aged 15 to 45 years, with a steadily increasing incidence worldwide [1]. Although advances in diagnosis and therapy have led to five-year survival rates exceeding 95% in early-stage disease [1-4], treatment-related consequences, particularly those involving body image, remain clinically relevant but often underestimated. Radical orchiectomy, the standard first-line therapy for localized disease, is curative in most cases; however, the anatomical and symbolic loss of a testicle may profoundly affect male identity, sexuality, and self-perception, particularly in cultural contexts where masculinity is closely associated with genital integrity [1,4].

Testicular prosthesis implantation has been proposed as a simple, safe, and reconstructive strategy to mitigate these psychosocial effects. Previous studies have shown high satisfaction rates ranging from 77% to 97% [2,3]. In a Portuguese cohort, 97.7% of recipients reported their prosthesis as “excellent” or “good,” and over 88% expressed willingness to undergo the procedure again [2]. Despite these encouraging results, the evidence regarding its impact on global self-esteem remains mixed. While some studies report improvement in body image or sexual confidence [4], others, such as Catanzariti et al., found no statistically significant change in Rosenberg Self-Esteem Scale scores after implantation, suggesting that prostheses may improve localized perceptions without necessarily affecting overall self-worth [4].

Moreover, dissatisfaction with specific implant features remains common: up to 44% of patients describe excessive firmness, 20% to 40% report high positioning, and approximately one-third express discontent with implant size [1]. Nonetheless, global satisfaction tends to remain high, and a majority of men would still recommend the procedure [2,5].

Despite its potential benefits, prosthesis counseling is inconsistently offered. Only two-thirds of patients report receiving formal information on testicular implants, and nearly one-third perceive such counseling as insufficient or rushed [1]. This variability in access and information reflects a broader issue of inequity in aesthetic reconstruction within oncologic care. In Mexico and across Latin America, the literature on this topic is notably scarce, and the few available studies have focused primarily on pediatric populations [6]. To date, very limited peer-reviewed data have described the psychosocial impact of testicular prosthesis among adult Latin American survivors of testicular cancer.

In this context, the present study seeks to address a critical evidence gap by evaluating the relationship between testicular prosthesis placement and self-esteem among patients undergoing radical orchiectomy. By comparing psychosocial outcomes between patients with and without prostheses and exploring satisfaction dimensions in recipients, we aim to inform clinical decision-making and advocate for more equitable, patient-centered approaches to oncologic rehabilitation.

## Materials and methods

We conducted a retrospective, observational, comparative study to evaluate the association between testicular prosthesis placement and psychosocial outcomes, specifically self-esteem and body satisfaction, among patients with non-metastatic testicular cancer who underwent radical orchiectomy. The study was performed at the Hospital General de México “Dr. Eduardo Liceaga,” a high-volume tertiary care center in Mexico City, and included patients treated between January 2019 and December 2024.

We identified all adult male patients (≥18 years) diagnosed with non-metastatic testicular cancer who underwent unilateral or bilateral radical orchiectomy within the study period. Eligible cases were identified through institutional surgical records and pathology databases. Inclusion required complete clinical documentation, availability of operative reports confirming whether a testicular prosthesis was placed, and successful completion of a follow-up telephone survey. We excluded patients with evidence of metastatic disease at the time of surgery, prior psychiatric diagnoses interfering with body image perception, congenital scrotal anomalies, or incomplete clinical records. Patients who could not be reached after multiple contact attempts or who declined participation were also excluded.

The primary exposure variable (non-randomized intervention) was the placement of a testicular prosthesis at the time of orchiectomy. Based on this intervention, participants were stratified into two comparison groups: the prosthesis group, which included patients who received a silicone-based testicular prosthesis during orchiectomy, and the control group, which consisted of patients who did not undergo prosthesis placement. The timing of the intervention was uniform, with all implants placed immediately following orchiectomy during the same surgical procedure. Radical orchiectomy with immediate testicular prosthesis placement was performed according to institutional practice. Prostheses were inserted through a scrotal approach with the creation of a dartos pouch. In all cases, the implant was positioned within the pouch and secured to the dartos fascia at the inferior pole to reduce migration. No additional fixation techniques were used. Prosthesis size was selected intraoperatively based on the estimated volume of the contralateral testis, without formal preoperative sizing protocols or patient-directed size selection.

All patients undergoing radical orchiectomy were systematically counseled preoperatively regarding the option of immediate testicular prosthesis placement. Eligibility for prosthesis implantation was based on the absence of local infection or surgical contraindications. In this public-sector setting, the cost of prostheses was covered by the institutional health system and did not represent an out-of-pocket expense for patients. The final decision to proceed with prosthesis placement was made jointly by the patient and treating surgeon following counseling, according to the patient's preferences. All testicular prostheses were commercially available saline-filled implants of a single manufacturer and model. Prosthesis size selection and placement technique are described above. Standard perioperative antibiotic prophylaxis was administered according to institutional protocol. Postoperatively, patients received routine scrotal support and standard follow-up, without prosthesis-specific restrictions or interventions.

The primary outcome was self-esteem, operationalized through the Rosenberg Self-Esteem Scale (RSES), a 10-item validated instrument yielding scores from 0 to 30 or 0 to 40, with higher scores reflecting greater global self-esteem [7]. The RSES was administered via structured telephone interviews by trained personnel using a standardized script to reduce interviewer bias. The survey was administered at a median of six months (IQR: 3-8) following orchiectomy. Self-esteem was assessed using the Spanish-language version of the Rosenberg Self-Esteem Scale, which has been previously validated in Spanish-speaking populations. No additional cultural or linguistic adaptations were performed for this study. Internal consistency reliability was not formally reassessed in the present sample.

Secondary outcomes, assessed exclusively in the prosthesis group, included body satisfaction dimensions (size, weight, shape, position, consistency), discomfort during physical or sexual activity, and subjective willingness to repeat or recommend the procedure. These were captured via a structured satisfaction questionnaire developed by the research team and piloted internally for comprehensibility. A study-specific satisfaction questionnaire was developed by the research team to assess patient perceptions following prosthesis placement (Appendix 1). Items were generated to cover cosmetic appearance, firmness, position, comfort, and overall satisfaction, and were reviewed for face clarity prior to use. The instrument employed a five-point Likert-type response scale. Formal psychometric validation was not conducted, limiting the interpretability of secondary outcomes. Incomplete information on non-responders precluded formal comparison between responders and non-responders and limited assessment of potential response bias; therefore, multiple imputation methods were not feasible.

From a total cohort of 110 patients identified during the study period, 92 met the inclusion criteria and completed follow-up. Of these, 41 had received a testicular prosthesis, and 51 did not. Although no formal a priori sample size calculation was conducted, exploratory post hoc power estimation revealed that the final sample size provided >80% power to detect a standardized mean difference of ≥0.7 in Rosenberg scores between groups, assuming a two-sided α = 0.05.

Demographic, clinical, surgical, and histopathological data were extracted from electronic and paper medical records by the principal investigator. Surgical laterality, operative time, tumor histology (seminomatous vs. non-seminomatous), and postoperative complications (classified according to Clavien-Dindo grades I-II) were recorded [8]. Survey data were independently entered into a secure IBM SPSS Statistics for Windows, Version 25 (Released 2017; IBM Corp., Armonk, New York, United States) database and validated by duplicate entry and audit verification.

All analyses were performed using IBM SPSS Statistics for Windows, Version 25. A two-sided alpha level of 0.05 was used for all analyses. Continuous variables were compared using appropriate parametric or nonparametric tests based on data availability, and exact tests were applied when cell counts were small. Categorical variables were summarized using frequencies and proportions. Between-group comparisons for continuous variables were performed using Student’s t-test (for normally distributed data) or the Mann-Whitney U test (for non-normal data), with effect size estimates and 95% confidence intervals. Categorical variables were compared using chi-square tests or Fisher’s exact tests where appropriate.

The primary comparison of interest was the mean RSES score between the prosthesis and control groups. A two-sided p-value <0.05 was considered statistically significant. Missing data were minimal (<5%) and were handled using complete case analysis; no imputation was performed.

This study was reviewed and approved by the Institutional Research Ethics Committee of the Hospital General de México (approval number: HGM-02-0003, date of approval: January 15, 2024). Given the retrospective nature of the study, written informed consent was waived for the chart review phase. However, verbal informed consent was obtained prior to telephone survey administration. All data were anonymized and handled in accordance with the General Law for the Protection of Personal Data in Mexico.

## Results

Patient characteristics

A total of 110 patients underwent radical orchiectomy for non-metastatic testicular cancer between January 2019 and December 2024. Of these, 92 patients (83.6%) completed the structured telephone follow-up and were included in the final analysis. Among them, 41 patients (45%) received a testicular prosthesis during orchiectomy, and 51 patients (55%) did not.

Baseline demographic, oncologic, and perioperative characteristics were well-balanced between groups and are summarized in Table 1. Mean age did not differ significantly between the prosthesis and control groups (31.2 ± 5.8 vs. 30.4 ± 6.1 years; t = 0.74, df = 90, p = 0.46). Histological distribution was comparable, with no significant between-group difference in the proportion of seminomatous versus non-seminomatous germ cell tumors (χ² = 0.21, p = 0.79) or in surgical laterality (χ² = 1.38, p = 0.50). The proportion of bilateral procedures was numerically higher in the prosthesis group (7% vs. 2%), though this difference did not reach statistical significance (Fisher's exact test, p = 0.36). As expected, median operative time was significantly longer in the prosthesis group (48 min (IQR: 42-55) vs. 37 min (IQR: 31-44); U = 614, p < 0.001), consistent with the additional procedural steps required for implant placement. Postoperative complication rates were low and did not differ significantly between groups (7% vs. 6%; Fisher's exact test, p = 0.86). Observed complications included minor scrotal hematoma and self-limited seroma, all managed conservatively, with no infectious events, prosthesis extrusion, or reinterventions recorded.

**Table 1:** Baseline characteristics of patients undergoing radical orchiectomy with and without testicular prosthesis *NSGCT: non-seminomatous germ cell tumor

Variable	No Prosthesis (n = 51)	Prosthesis (n = 41)	Total (n = 92)
Mean age, years	30	31	30.5
Laterality			
Left	25 (49%)	21 (51%)	46 (50%)
Right	25 (49%)	17 (42%)	42 (46%)
Bilateral	1 (2%)	3 (7%)	4 (4%)
Histological subtype			
Seminoma	25 (49%)	19 (46%)	44 (48%)
NSGCT*	24 (48%)	20 (49%)	44 (48%)
Others	2 (3%)	2 (5%)	4 (4%)
Median surgical time, minutes	37	48	—
Postoperative complications			
Clavien–Dindo I–II	3 (6%)	3 (7%)	6 (7%)

Satisfaction with prosthesis characteristics

Among the 41 prosthesis recipients, overall satisfaction was reported by 100% of participants, and 98% described the implant as comfortable. Dimension-specific satisfaction ratings are presented in Table 2. Notably, firmness was the most frequent source of dissatisfaction, with 41% of recipients perceiving the implant as too firm. In terms of behavioral intent, 96% of patients stated they would choose to undergo the procedure again, and 100% would recommend it to others. Only one patient (2%) reported pain during physical activity, and one reported discomfort during sexual intercourse (Table 3).

**Table 2:** Patient-reported satisfaction with testicular prosthesis characteristics (n = 41)

Characteristic	Rating	n (%)
Size	Very small	1 (2.5)
	Appropriate	39 (95.0)
	Very large	1 (2.5)
Weight	Appropriate	39 (95.0)
	Not appropriate	2 (5.0)
Shape	Appropriate	35 (85.4)
	Not appropriate	6 (14.6)
Position	Appropriate	29 (70.7)
	Not appropriate	12 (29.3)
Consistency	Soft	4 (9.8)
	Appropriate	20 (48.8)
	Firm	17 (41.5)

**Table 3:** Prosthesis-related adverse perceptions among recipients (n = 41) *“Yes” indicates the presence of the reported symptom or impact.

Question	Yes n (%)*	No n (%)
Pain associated with the prosthesis	3 (7%)	38 (93%)
Negative impact on physical activity	1 (2%)	40 (98%)
Negative impact on sexual activity	1 (2%)	40 (98%)

Self-esteem outcomes

The primary outcome of interest, global self-esteem, was significantly higher in the prosthesis group compared to the control group. The mean Rosenberg self-esteem score was higher in patients with a prosthesis compared with those without (31.8 ± 4.1 vs. 23.6 ± 5.2; mean difference 8.2 points, 95% CI 4.3-12.1; p = 0.003). No significant adverse outcomes or safety concerns related to prosthesis use were identified.

## Discussion

Immediate testicular prosthesis implantation following radical orchiectomy was associated with higher self-esteem, as measured by the RSES, compared to patients without a prosthesis. This finding is consistent with the hypothesis that anatomical restoration mitigates the psychosocial burden of testicular loss, a critical consideration given the high survival rates in localized testicular cancer and its impact on body image and masculine identity, particularly in young men [4,7-9]. Our results extend prior evidence by demonstrating a robust between-group difference in a public-sector setting, addressing a gap in the literature for Latin American populations.

Patients with a prosthesis reported high satisfaction levels: 100% expressed global satisfaction, 95% with size and weight, 85% with shape, and 71% with anatomical position. These findings are congruent with contemporary studies reporting high acceptance of modern silicone-based testicular prostheses [8,3]. However, 41% of recipients perceived the prosthesis as “too firm,” and 29% expressed dissatisfaction with its scrotal position, mirroring recurrent device-specific complaints and highlighting opportunities for design optimization [10,11]. Despite these focal dissatisfactions, behavioral endorsement remained strong: 96% of patients would choose to undergo the procedure again, and 100% would recommend it to others, reinforcing the net positive value of reconstruction [9,10].

Functional tolerability was high, with negligible rates of prosthesis-related pain or interference with physical and sexual activity (Table 3). Strong behavioral endorsement, with nearly all recipients willing to repeat the procedure and to recommend it to others, reinforces the net positive value of immediate reconstruction, as reported by Ramos et al. [2] and Araújo et al. [3]. These results are consistent with prospective data confirming that implantation is a safe procedure with negligible effects on sexual function [4]. Qualitative evidence further suggests that recipients progressively normalize the presence of the implant over time, integrating it into their body image [11]. These findings indicate that intraoperative implantation offers meaningful psychosocial benefits with minimal procedural risk.

The 45% acceptance rate among eligible patients, higher than historical uptake in population-based cohorts, aligns with reports that systematic offering of prostheses enhances adoption [11,12]. This suggests that consistent counseling could address barriers to access, as inadequate counseling has been identified as a contributor to underuse despite high satisfaction among recipients [7,13]. While our study did not directly assess the role of preoperative counseling, the observed outcomes underscore the importance of integrating such discussions into routine care.

Despite existing literature on testicular prosthesis outcomes, evidence from public-sector Latin American settings remains limited. Our study adds comparative data between men who did and did not receive a prosthesis within a real-world Mexican survivorship context, highlighting high acceptability and low complication burden.

From a health-system perspective, these findings highlight the importance of equitable access to testicular prosthesis placement within public-sector settings. Standardized preoperative counseling may help ensure that all patients receive consistent information regarding prosthesis options and potential psychosocial implications, independent of provider or institutional variability. In publicly funded health systems such as those in Mexico and much of Latin America, coverage of prosthesis costs may reduce financial barriers and support informed, preference-sensitive decision-making. Incorporating structured counseling and clear reimbursement pathways could improve equity and consistency in survivorship care.

Our findings suggest that immediate testicular prosthesis placement is associated with higher self-esteem and high levels of patient satisfaction following orchiectomy. These results align with previous studies reporting favorable psychosocial outcomes in selected populations. In a recent retrospective study by Boeri et al. (2025), patients who received a testicular prosthesis reported higher satisfaction, lower decision regret, and better body image compared with those who declined implantation, who more frequently experienced persistent discomfort, shame, and partner dissatisfaction. Together, these findings underscore the importance of comprehensive preoperative counseling that addresses both patient expectations and potential relational factors [13].

Limitations of this study include its retrospective design, the absence of baseline self-esteem scores prior to orchiectomy, the use of a non-validated satisfaction instrument developed by the research team, and a single-timepoint follow-up, which precludes assessment of outcome durability. Psychosocial responses to orchiectomy and prosthesis placement may evolve over time, and early or delayed assessments could yield different self-esteem or satisfaction profiles. Residual confounding by unmeasured psychological factors is also possible. Nevertheless, the consistency across self-esteem, satisfaction, and behavioral intent strengthens the inference that prosthesis placement contributes materially to psychosocial recovery. Relationship status was not systematically collected, and therefore, its potential influence on prosthesis decision-making, body image, or self-esteem could not be evaluated. No multivariable adjustment was performed, limiting control of potential confounders. In addition, no data were collected regarding partner involvement or partner attitudes toward prosthesis placement, which may represent an unmeasured factor influencing patient satisfaction or emotional adjustment. Educational attainment was also not collected, and therefore, its potential impact on health literacy, understanding of the procedure, or engagement with preoperative counseling could not be assessed.

## Conclusions

Immediate implantation of a testicular prosthesis at the time of radical orchiectomy was associated with substantially higher self‑esteem, high multidimensional satisfaction, and minimal adverse impact. Routine preoperative counseling and offering of prosthesis placement should be incorporated into testicular cancer survivorship care to optimize psychosocial recovery. Prospective multicenter studies with longitudinal follow-up are warranted to confirm the durability of the benefit and refine patient selection and counseling strategies.

## Appendices

Appendix 1 

**Table 4:** Structured satisfaction questionnaire for testicular prosthesis recipients

Item	Domain	Question / Statement	1	2	3	4	5
Domain 1: IMPLANT APPEARANCE							
Response options for this domain:			1 Very Dissatisfied	2 Dissatisfied	3 Neutral	4 Satisfied	5 Very Satisfied
1	Implant Appearance	The size of the prosthesis matches my natural testicle satisfactorily.	☐	☐	☐	☐	☐
2	Implant Appearance	The weight of the prosthesis feels natural within the scrotum.	☐	☐	☐	☐	☐
3	Implant Appearance	The shape of the prosthesis resembles that of a natural testicle.	☐	☐	☐	☐	☐
4	Implant Appearance	The position of the prosthesis within the scrotum appears anatomically appropriate.	☐	☐	☐	☐	☐
5	Implant Appearance	The firmness (consistency) of the prosthesis feels natural.	☐	☐	☐	☐	☐
Domain 2: PHYSICAL COMFORT							
Response options for this domain:			1 Never	2 Rarely	3 Sometimes	4 Often	5 Always
6	Physical Comfort	I experience discomfort or pain in the scrotal area at rest.	☐	☐	☐	☐	☐
7	Physical Comfort	I experience discomfort or pain during physical activity (e.g., exercise, sports).	☐	☐	☐	☐	☐
8	Physical Comfort	The prosthesis interferes with wearing tight clothing or underwear.	☐	☐	☐	☐	☐
Domain 3: FUNCTIONAL IMPACT							
Response options for this domain:			1 Never / Strongly Disagree	2 Rarely / Disagree	3 Sometimes / Neutral	4 Often / Agree	5 Always / Strongly Agree
9	Functional Impact	The presence of the prosthesis negatively affects my sexual activity. *	☐	☐	☐	☐	☐
10	Functional Impact	I feel self-conscious about the prosthesis during intimate encounters. *	☐	☐	☐	☐	☐
11	Functional Impact	Over time, I have become accustomed to the presence of the prosthesis.	☐	☐	☐	☐	☐
Domain 4: GLOBAL EXPERIENCE							
Response options for this domain:			1 Strongly Disagree	2 Disagree	3 Neutral	4 Agree	5 Strongly Agree
12	Global Experience	Overall, I am satisfied with the result of the testicular prosthesis.	☐	☐	☐	☐	☐
13	Global Experience	The prosthesis has contributed positively to my self-image and confidence.	☐	☐	☐	☐	☐
14	Global Experience	The prosthesis has helped me cope with the emotional impact of orchiectomy.	☐	☐	☐	☐	☐
Domain 5: BEHAVIORAL INTENT							
Response options for this domain:			1 Definitely Not	2 Probably Not	3 Unsure	4 Probably Yes	5 Definitely Yes
15	Behavioral Intent	If I could decide again, I would choose to receive the testicular prosthesis.	☐	☐	☐	☐	☐
16	Behavioral Intent	I would recommend testicular prosthesis placement to other patients undergoing orchiectomy.	☐	☐	☐	☐	☐
